# Mitochondrial uncoupling and the disruption of the metabolic network in hepatocellular carcinoma

**DOI:** 10.18632/oncotarget.27680

**Published:** 2020-08-04

**Authors:** Lilia Turcios, Francesc Marti, David S. Watt, Lilia M. Kril, Aman Khurana, Fanny Chapelin, Chunming Liu, Joseph B. Zwischenberger, B. Mark Evers, Roberto Gedaly

**Affiliations:** ^1^Department of Surgery, Transplant Division, College of Medicine, University of Kentucky, Lexington, KY, USA; ^2^Department of Molecular and Cellular Biochemistry, College of Medicine, University of Kentucky, Lexington, KY, USA; ^3^Lucillle Parker Markey Cancer Center, College of Medicine, University of Kentucky, Lexington, KY, USA; ^4^Department of Biomedical Engineering, College of Medicine, University of Kentucky, Lexington, KY, USA; ^5^Department of Radiology, College of Medicine, University of Kentucky, Lexington, KY, USA

**Keywords:** hepatocellular carcinoma, proton uncouplers, Wnt/β-catenin pathway, glutamine metabolism, mitochondria

## Abstract

Background: Hepatocellular Carcinoma (HCC) is the third most common cause of cancer related death worldwide. Adequate treatment options for patients with advanced HCC are currently limited.

Materials and Methods: We studied the anti-HCC effect of FH535 and a novel derivative Y3, on proliferation, mitochondrial function and cellular metabolism focusing on the three key substrates, glutamine, glucose, and fatty acids.

Results: FH535 and Y3 disrupted mitochondrial redox control in HCC cells that resulted from uncoupling mechanisms that increased proton leakage and decreased ATP production leading to apoptosis. The uncoupling effects of the sulfonamides in HCC cells were supported by the loss of activity of the methylated analogs. The accumulation of ROS significantly contributed to cell damage after the impaired autophagic machinery. These sulfonamides, FH535 and Y3, targeted glutamine and fatty acid metabolism and caused HCC cell reprograming towards the preferential use of glucose and the glycolytic pathway.

Conclusions: FH535, and Y3, demonstrated potent anti-HCC activity by targeting OXPHOS, increasing dangerous levels of ROS and reducing ATP production. These sulfonamides target glutamine and FA metabolic pathways significantly increasing the cellular dependency on glycolysis.

## INTRODUCTION

Hepatocellular carcinoma (HCC) is the most common primary liver cancer with more than 800,000 new cases annually [1]. Surgery either liver resection or transplantation are considered the treatment of choice in the early stages of HCC. Regrettably, most patients present with advanced stages of the disease for which limited treatment options result in dismal 5-year survival in the range of 10% according to the American Cancer Society. In recent years, sorafenib, a known multi-kinase inhibitor, became the first-line treatment for advanced-stage HCC, but unfortunately provided limited improvement in patient survival [2]. Although other kinase and immune checkpoint inhibitors are now considered second-line options, the data supporting improved outcomes relative to sorafinib is not yet compelling [3, 4].

Because to the lack of treatment options in advanced cases, our group and others have developed strategies using small-molecules targeting different pathways with or without immunotherapy for late stages in pre-clinical or clinical studies [3–11]. The Wnt/β-catenin pathway is critical for HCC cell proliferation, progression and stemness, and this pathway is aberrant in 30–50% of the HCC tumors [12–16]. As a consequence, the Wnt/β-catenin pathway represents an attractive molecular target for HCC treatment. In recent years, we reported that 2,5-dichloro-N-(2-methyl-4-nitrophenyl) benzenesulfonamide (FH535), a sulfonamide that targets this pathway and alters mitochondrial respiration and the autophagic process, is active alone and in combination with other drugs against HCC *in vitro* and *in vivo* [17]. We have synthesized new FH535 derivatives with improved the anti-HCC potency/activity that are now being tested in other cancers. We recently reported that FH535 affected the Wnt/β-catenin pathway functioning as a mitochondrial “proton uncoupler” in colon cancer cells. Recent reports are assessing the therapeutic opportunities of targeting mitochondria uncoupling activity in cancer. A study by Alasadi et al. used the mitochondrial uncouplers, Niclosamide Ethanolamine and Oxyclozanide, on metastatic colorectal cancer [18]. Another group studied how sorafenib, a drug commonly used in the treatment of advanced HCC, acts as a mitochondrial uncoupler at low doses [19]. We demonstrated that the ability of FH535 to transport protons from the mitochondrial intermembrane space into the mitochondrial matrix altered the transmembrane potential and disrupted ATP generation [20, 21]. We now report a study of the functional link between the uncoupler activities of FH535 and a new analog of FH535, 2,5-dichloro-*N*-(4-nitronaphthalen-1-yl) benzenesulfonamide (Y3) and the methylated analogs (FH535-me) and Y3 (Y3-me) on HCC activity.

## RESULTS

### Cell proliferation and apoptosis

**Figure 1:**
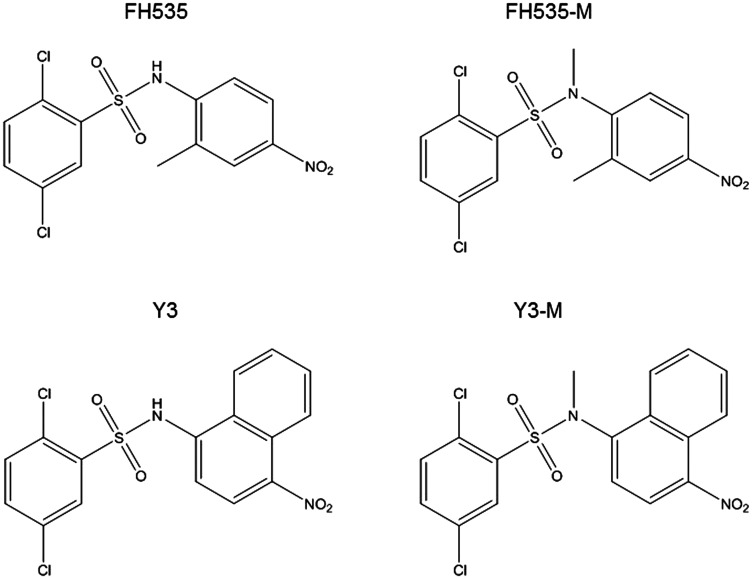
Structures of FH535 and Y3 and methylated compounds.

**Figure 2:**
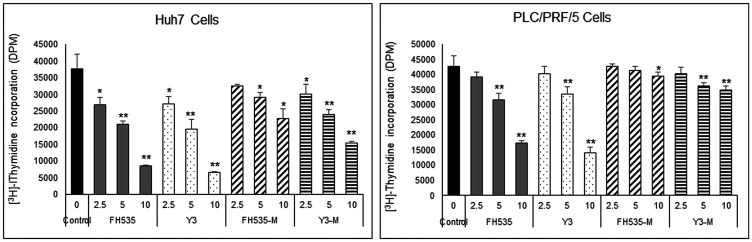
Effect of FH535 and Y3 Methylated derivatives on HCC cell proliferation. Cell proliferation was measured on Huh7 and PLC/PRF/5 cells using [^3^H]-thymidine incorporation assay after 72 h treatment with FH535, Y3 or their methylated analogs at the concentrations indicated. Results are represented as mean ± SD, *n* = 4. ^*^ *p* ≤ 0.05, ^**^ *p* ≤ 0.001 respect to vehicle treated cells.

**Figure 3:**
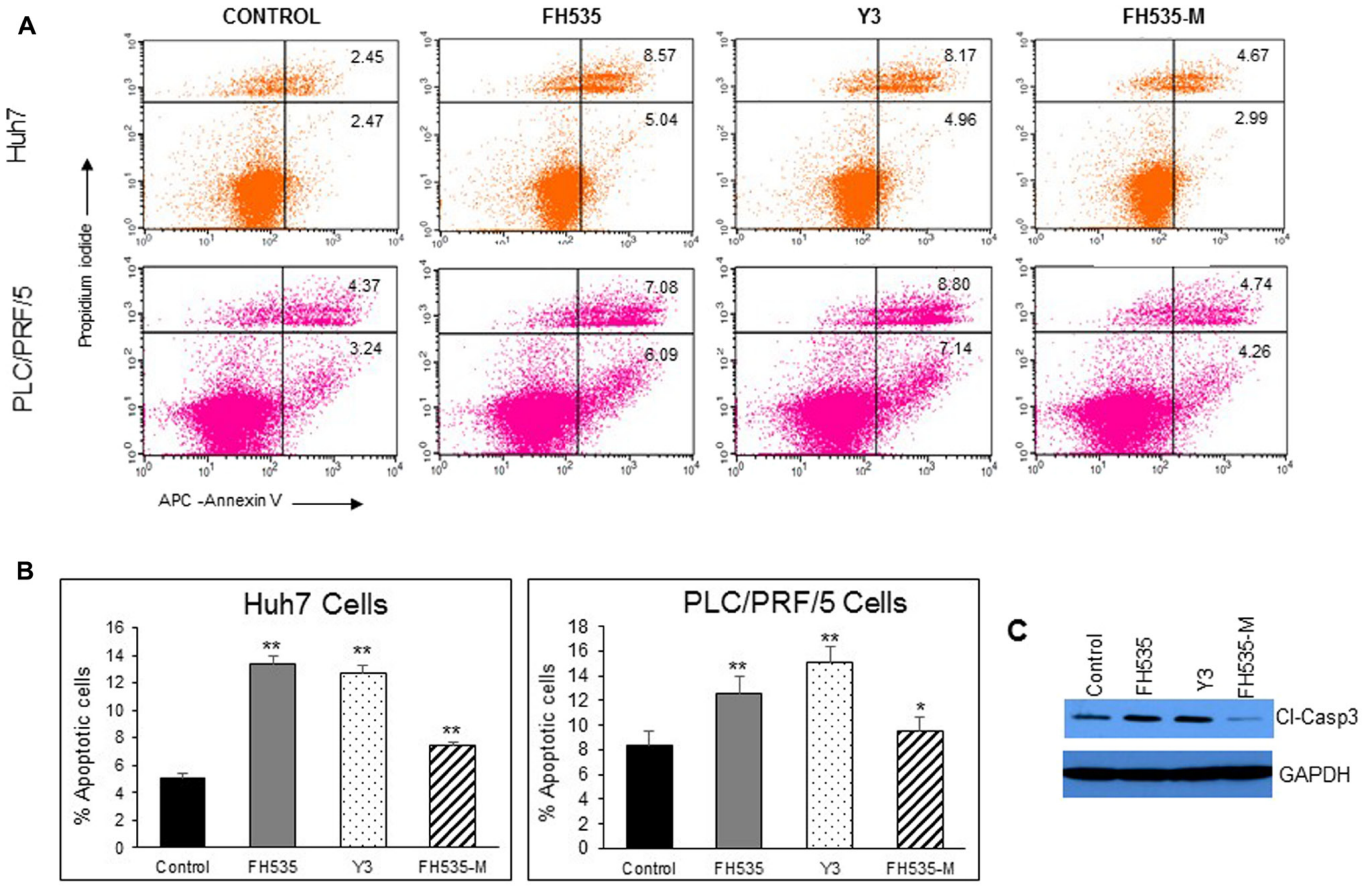
Analysis of apoptosis by Annexin V-APC/propidium iodide (PI) double staining of HuH7 and PLC/PRF/5 cells after 48 h treatment with vehicle or 10 μM of FH535, Y3 and FH535-M. (**A**) Two-color flow cytometry dot plots show the percentages of living cells as negative for both annexin V and PI; early-stage apoptotic cells as the populations testing Annexin V positive and PI negative, and late-stage apoptotic/necrotic cells as double-positive cells. Results are represented in (**B**) as percentages ± SD, *n* = 3. ^**^ *p* ≤ 0.001; (**C**) Detection of Caspase 3 expression by western blot after treatment of Huh7 cells with vehicle control or 10 μM of FH535, Y3, or FH535-M for 48 h.

We previously demonstrated that FH535 and its analog Y3 inhibited the proliferation of hepatoma cell lines, Huh7, PLC/PRF/5 and Hep3B Cells [17]. These sulfonamides altered mitochondrial respiration activity through increased proton transport from the inner membrane space to the mitochondrial matrix. This dissipation of the proton and associate charge gradient **Δ**ψm across the inner mitochondrial membrane reduced ATP production [20]. These effects on cell proliferation of the hepatoma cell lines were in agreement with a recent report in which the OXPHOS uncoupling activity of FH535 and Y3 were severely impaired after the substitution of the active hydrogen in the sulfonamide bond that was involved in the proton translocation with an N-methyl group in the sulfonamide bond (Figure 1). We also tested the effect of these methylated analogs, FH535-Me and Y3-Me, on the proliferation of the HCC cell lines Huh7 and PLC/PRF/5 and observed a significant loss of inhibitory function on cell proliferation compared with the corresponding non-methylated progenitors, FH535, and Y3 (Figure 2). The pro-apoptotic effect of FH535-Me was markedly reduced compared to FH535 (Figure 3), an outcome that linked proton translocation in the mitochondria to the inhibition of cell proliferation by FH535 and Y3.

### Mitochondrial respiration (OCR), Δψm, and ROS

**Figure 4:**
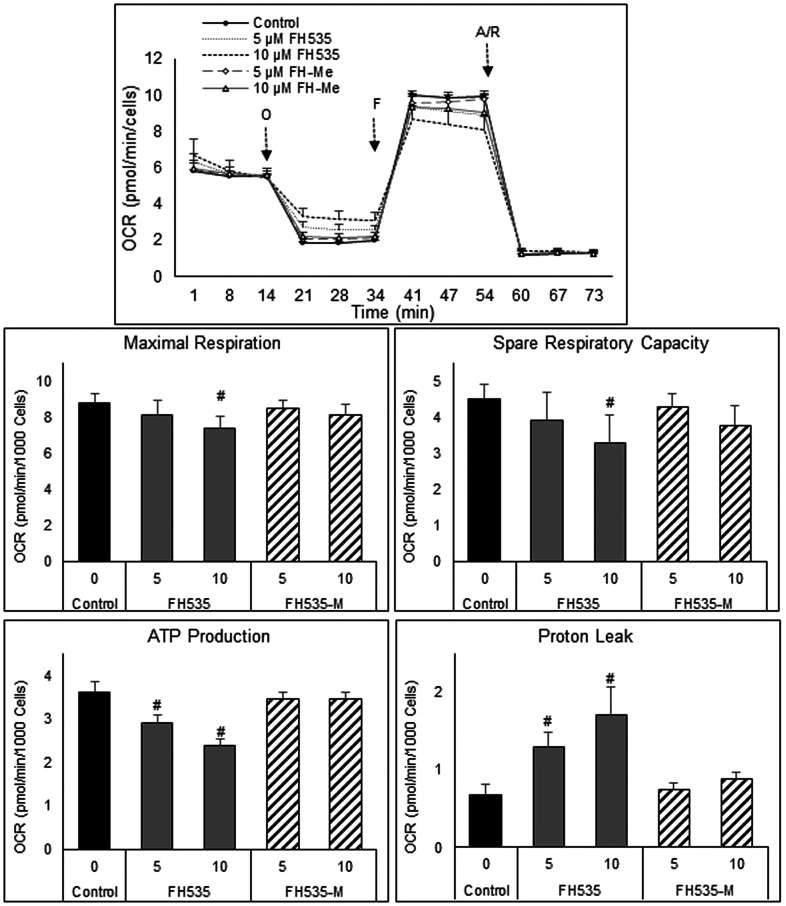
Mitochondrial respiration changes induced after treatment of HCC cells for 24 h with FH535 and its methylated derivative. Representative OCR profiles of Huh7 cells after addition of the ATP-synthase inhibitor Oligomycin (O). FCCP (F) collapses them and maximizes OCR levels, and the Antimycin/Rotenone (A/R) shut down the mitochondrial respiratory function. (B) The parameters of ATP turnover, proton leak, maximal respiration and spare respiratory capacity were calculated with the Seahorse report generation software. Data are shown as mean ± SEM, *n* = 6–8. ^#^ *p* < 0.0001 *vs.* control. Statistical comparisons were performed using one-way ANOVA and Dunnett’s multiple comparisons test and pairwise comparisons with Student’s *t* test using Graphpad.

**Figure 5:**
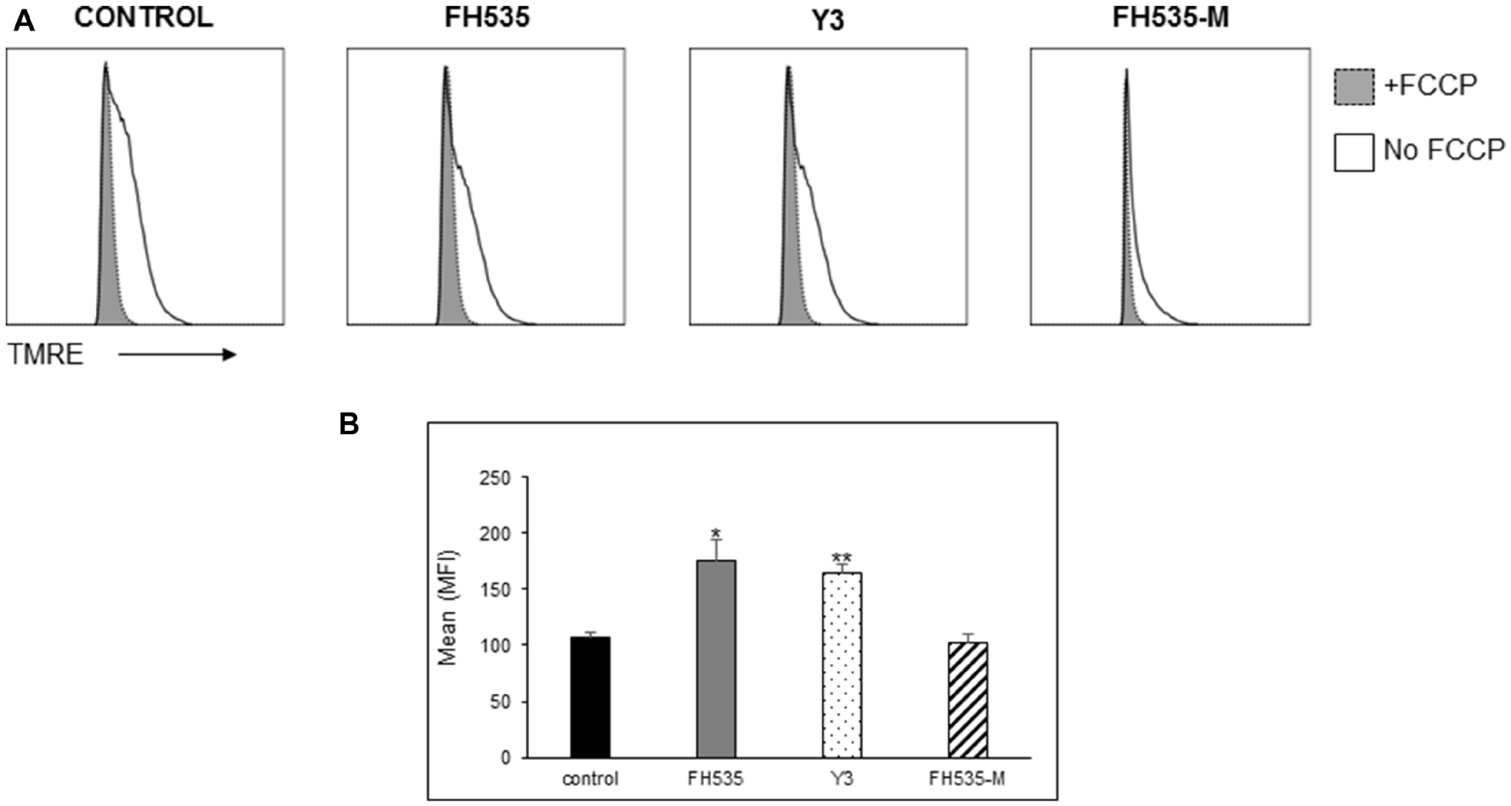
Effect of FH535 methylated compound on mitochondrial membrane potential (Δψm). After 48 h treatment with 10 μM of the indicated compound or DMSO vehicle control, Huh7 cells were labeled with the mitochondrial membrane potential dye TMRE and analyzed by flow cytometry. (**A**) Histograms depict the MFI of the TMRE fluorescence intensity of the samples treated with and without addition of the uncoupler FCCP (20 μM). (**B**) Quantification of the relative Δψm was determined by the TMRE MFI of FCCP-treated cells subtracted from basal TMRE MFI (*n* = 4 ± SD). ^**^ *p* < 0.0001 *vs.* control; NS = *p* > 0.05. Statistical comparisons were performed using one-way ANOVA and Dunnett’s multiple comparisons test and pairwise comparisons with Student’s *t* test.

**Figure 6:**
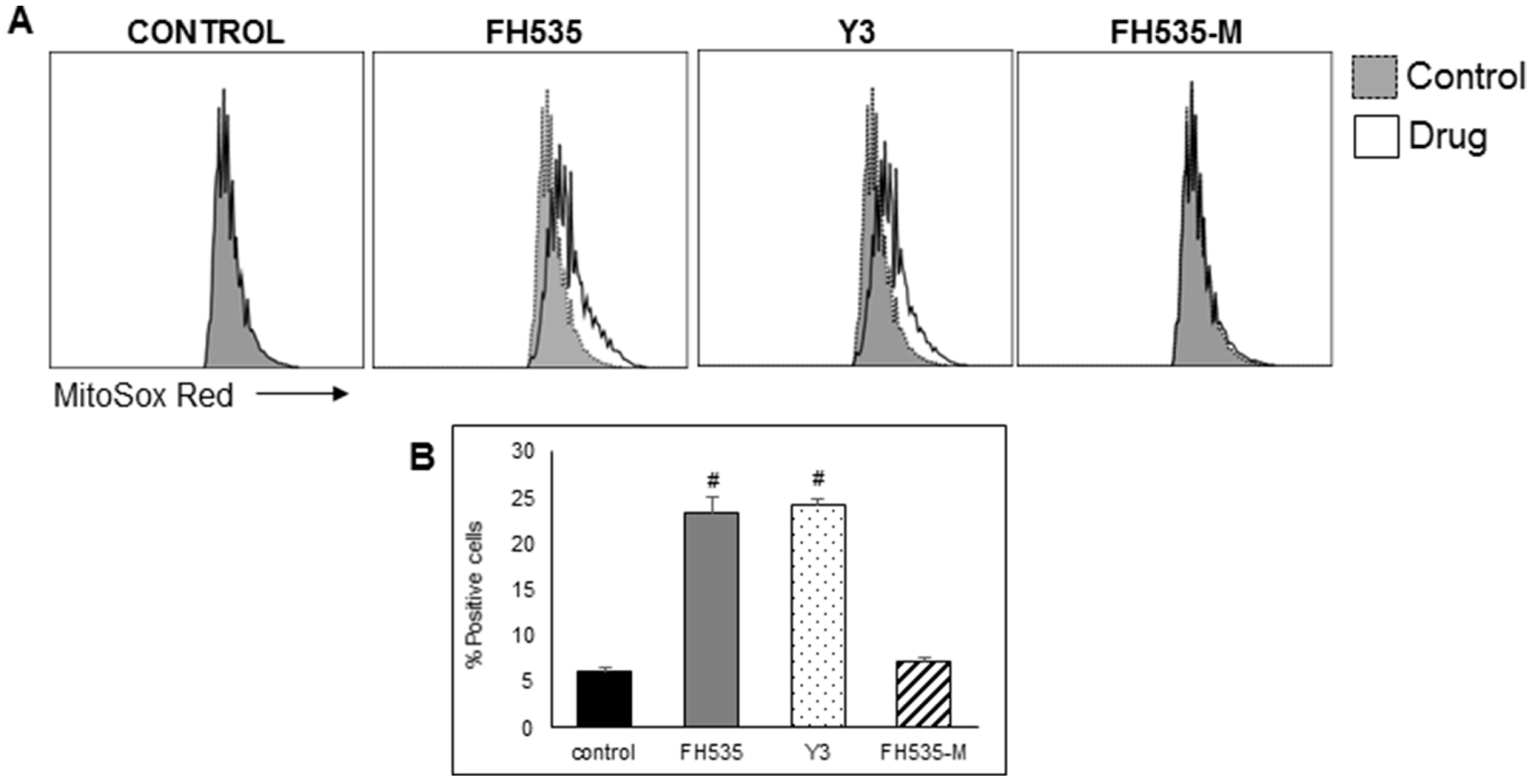
FH535 and its derivative Y3 increases mitochondrial superoxide generation in Huh7 cells. After 48 h treatment with DMSO control or 10 μM of the indicated compound, cells were labelled with MitoSOX red (5 μM) for 15 minutes and analyzed by flow cytometry. (**A**) Histogram representing the MFI of MitoSOX Red fluorescent intensity. (**B**) Quantification of percentage of positive cells ± SD, *n* = 3 ^#^ *p* < 0.0001 *vs.* control.

We next assessed the effect of FH535 methylation (*i.e*., FH535-Me) on mitochondrial respiration of Huh7 cells using the Agilent Seahorse XFe96 Cell Mito Stress test. We observed that FH535 and Y3 enhanced the proton leakage and significantly altered the maximal respiration capacity in Huh7 cells (Figure 4). These effects were lost in the methylated analogs, FH535-Me and Y3-Me, and consistent with **Δ**ψm measurements (Figure 5). The tightly regulated synthesis of mitochondrial ROS (mtROS) is essential for the maintenance of the cellular redox homeostasis. Excessive ROS generation will negatively affect proteins, lipids and DNA by oxidative damage, a process that can promote tumor initiation, growth and metastasis. FH535-uncoupling effects were not only linked to the changes in ETC activity and loss of **Δ**ψm, but also in mtROS levels. We observed that FH535, but not its methylated analog, FH535-Me, induced a significant accumulation of mtROS compared to control cells (Figure 6), an outcome that contributed to the anti-proliferative effect of FH535 in HCC cells.

### Effect in the glycolytic rate

Previous results demonstrated the synergistic inhibitory effect of the combination of FH535 and sorafenib on the proliferation of hepatoma cell lines and liver cancer stem cells [5, 9]. While FH535 preferentially targeted mitochondrial respiration, sorafenib preferentially decreased the glycolytic capacity of the cells. The synergistic actions of both drugs precluded cellular compensation for the energetic imbalance using either glycolysis or the mitochondrial ATP synthesis pathway as sources of ATP production [9].

**Figure 7:**
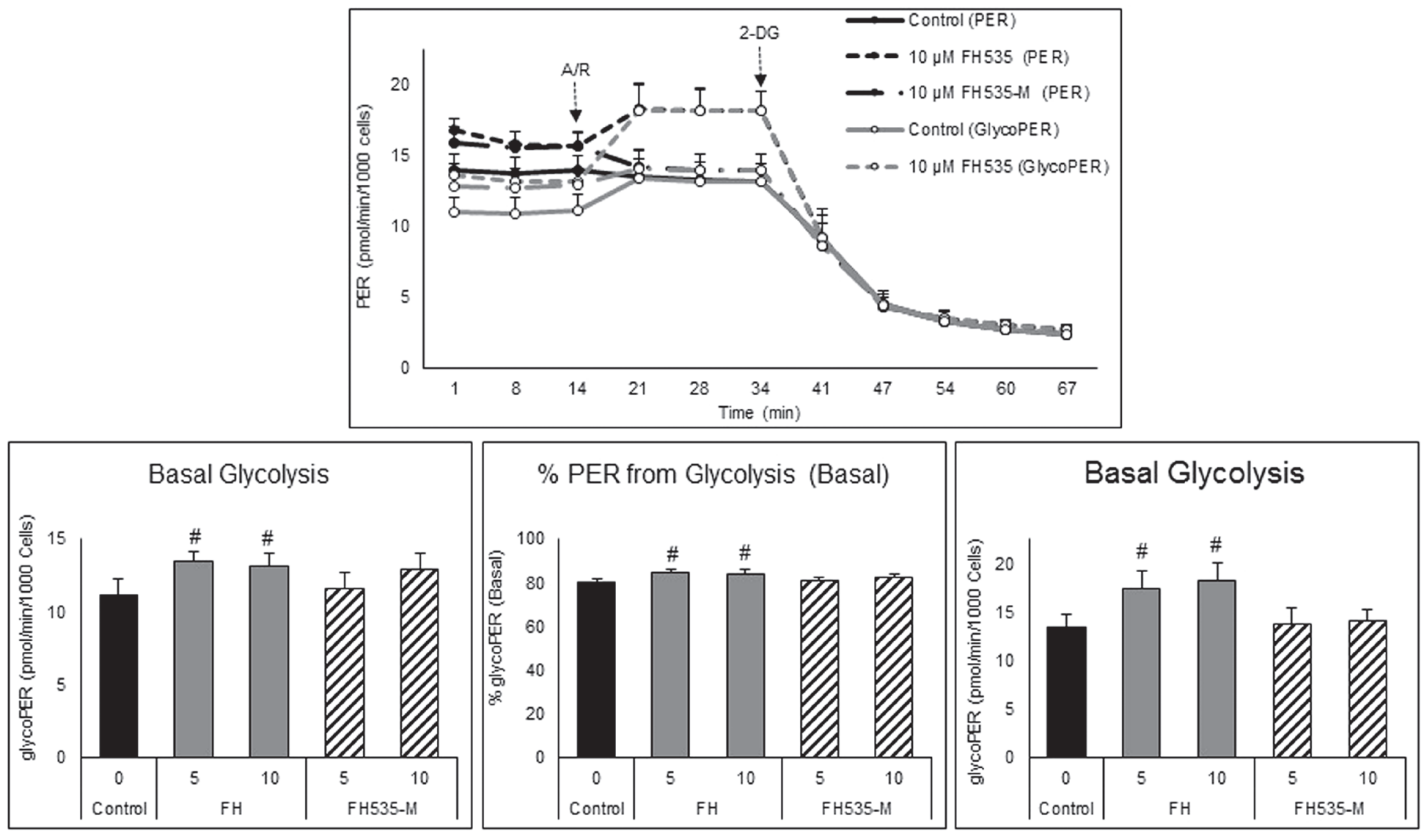
Effect of FH535 and FH535-M on the glycolytic rate of Huh7 cells. Total proton efflux rate (PER) and glycolytic proton efflux rate (glycoPER) were determined by measuring the OCR and ECAR under basal conditions and after sequential injections of rotenone/antimycin A (R/A, 1 μM each) and 2-DG (50 mM). The data were normalized to cell numbers and the proton efflux rate were calculated using the Seahorse Report Generator software. Data are shown as mean ± SEM, *n* = 6–8. ^#^ *p* < 0.0001 *vs.* control. Statistical comparisons were performed using one-way ANOVA and Dunnett’s multiple comparisons test and pairwise comparisons with Student’s *t* test using Graphpad.

We also investigated possible differences in the glycolytic activity induced by FH535 and FH535-Me using a Seahorse glycolytic rate assay. This assay distinguishes the amount of extracellular acidification (ECAR) produced by glycolysis in basal conditions from that originated by mitochondrial respiration, as well the compensatory glycolytic activity after inhibition of mitochondrial OXPHOS. As illustrated in Figure 7, cells treated with FH535 showed a significant increase in compensatory glycolysis that is not observed in cells treated with FH535-Me. These results indicated the metabolic “flexibility” of FH535-treated cells. Under high metabolic demand, FH535-treated cells, but not FH535-Me-treated cells, shifted towards increased glycolytic-dependent ATP production. In this context, the ability to increase the glycolytic rate using the glycolytic reserve capacity to compensate partially for loss of respiratory ATP production suggested the importance of glucose-driven, substrate-level phosphorylation in FH535-treated cells.

### Inhibition of glucose oxidation

**Figure 8:**
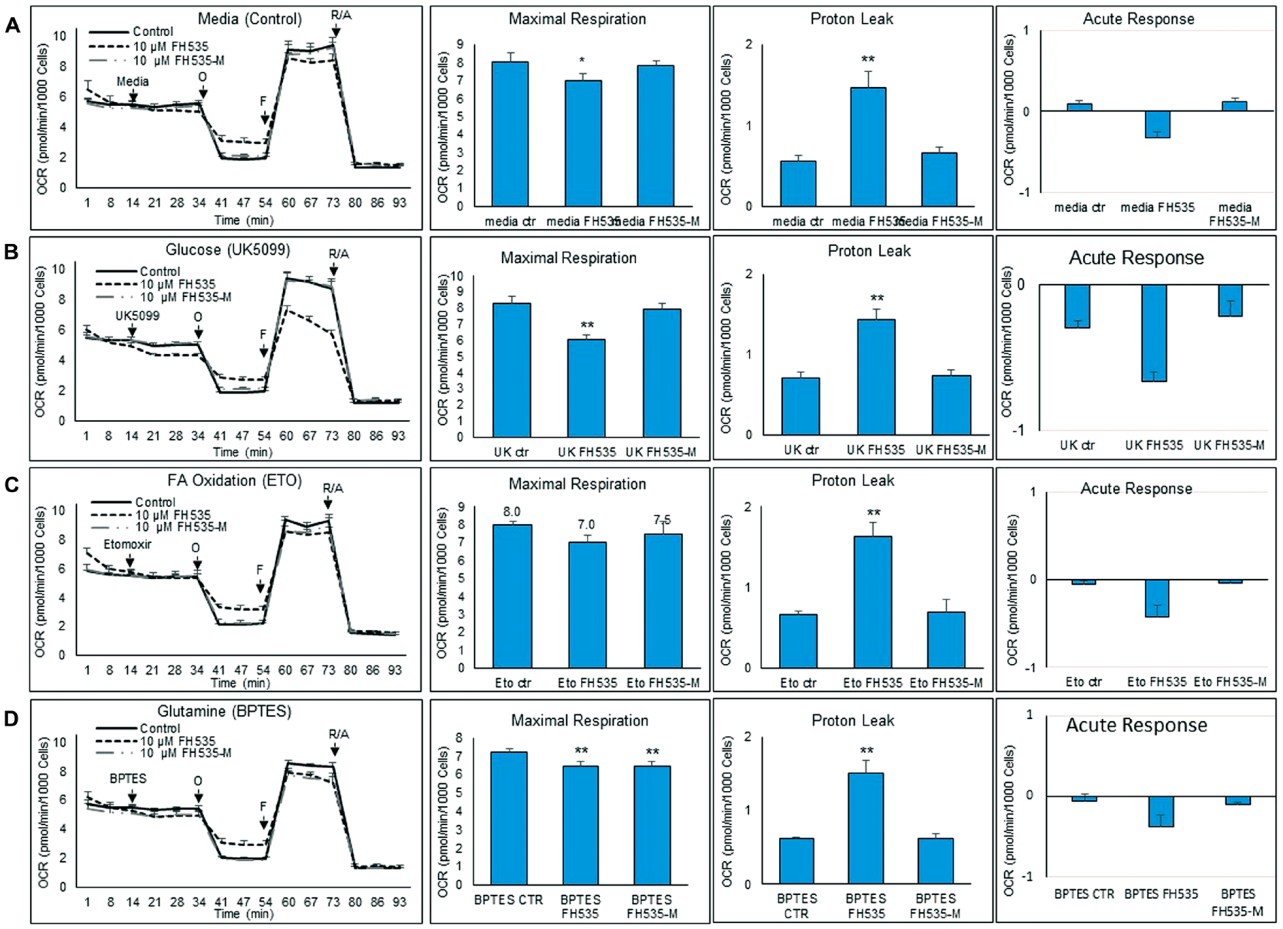
Changes in substrate utilization induced by FH535 and derivatives. Substrate oxidation analysis was performed in Huh7 cells after 24 h treatment with vehicle control or 10 μM indicated compound using a Seahorse substrate oxidation test. Left panels are representative of OCR profiles of Huh7 cells after addition of (**A**) media control, (**B**) UK5099, (**C**) Etomoxir (ETO), or (**D**) BPTES. Oligomycin (O), FCCP (F), and the Antimycin/Rotenone (A/R). The parameters of maximal respiration, proton leak and acute response were calculated with the Seahorse report generation software. Data are shown as mean ± SEM, *n* = 6–8. ^*^ *p* < 0.05 or ^**^ and *p* < 0.001 *vs.* control. Statistical comparisons were performed using one-way ANOVA and Dunnett’s multiple comparisons test and pairwise comparisons with Student’s *t* test using Graphpad.

To determine the induced FH535 changes on the utilization of metabolic substrates (*i.e*., glucose, LCFA and glutamine), we performed substrate oxidation assays using various inhibitors (Figure 8A–8D). To assess specifically the dependence on glucose oxidation, the UK5099 inhibitor was added to cells untreated (control) or treated with FH535 or FH535-Me. UK5099 prevented the transport of pyruvate into the mitochondria and its utilization as a TCA substrate. Cells treated with FH535 showed significantly lower maximal respiration levels than levels attainted using either FH535-Me or control (untreated) cells (Figure 8B). This decrease was more pronounced in FH535-treated cells injected with UK5099 than in FH535-treated cells with control medium and full availability of glucose; (Supplementary Figure 1B). This result indicates that mitochondrial OXPHOS underwent a substrate-preference shift in FH535-treated cells towards glucose-dependency that was driven by their inability to use alternative respiratory OXPHOS substrates.

### Inhibition of fatty acid oxidation

**Figure 9:**
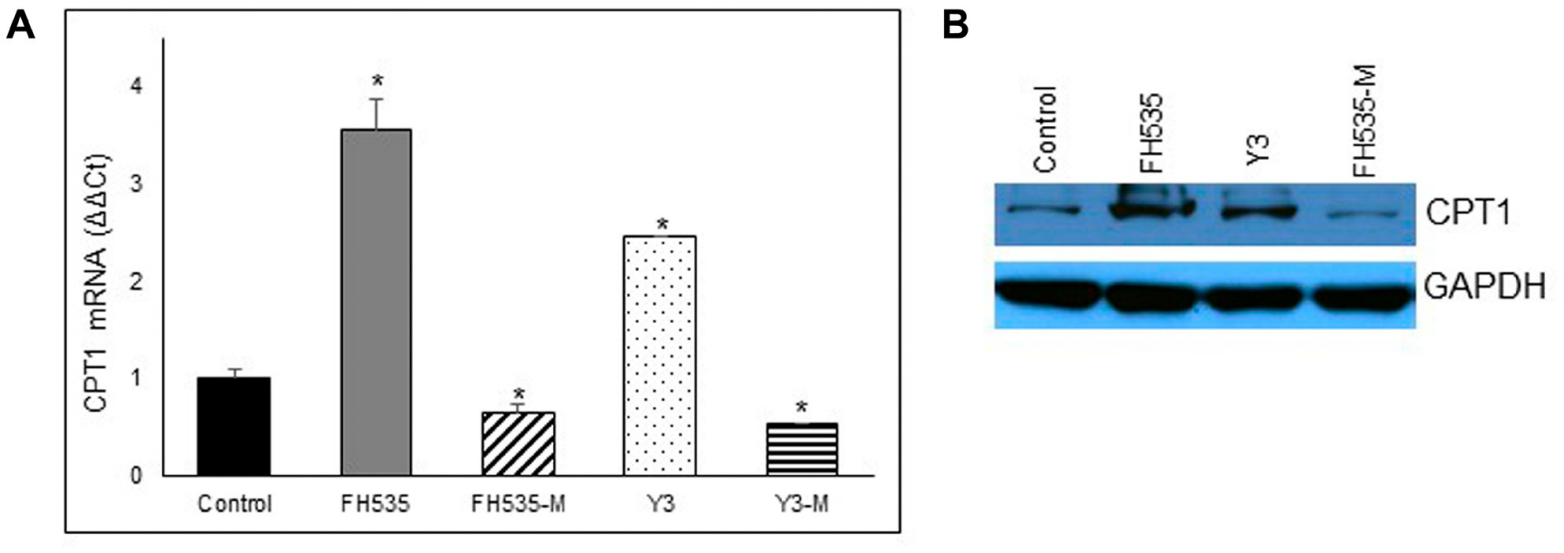
Effect of FH535 and derivatives on CPT1 expression. Expression of CPT1A in Huh7 cells was measured after 48 h treatment with 10 μM of indicated compound by real-time qPCR using B2M gene for normalization (**A**) and western blot (**B**). Values are represented as mean ± SD. ^*^ *p* < 0.05 *vs.* control.

We next assessed the effect of LCFA substrate oxidation inhibition in Huh7 cells by addition of etomoxir, a CPT1 inhibitor (Figure 8C). This enzyme is involved in the conjugation of LCFA to carnitine, a rate-limiting step in the transport of LCFA from cytoplasm into the mitochondria and, therefore, necessary in the β-oxidation of LCFA [22, 23]. We observed similar maximal respiration levels in FH535-treated cells with or without addition of etomoxir (Supplementary Figure 1C), an outcome indicating that the utilization of other substrates (glutamine and/or glucose) are not substantially altered by FH535 treatment and are equally responsive upon the inhibition of LCFA oxidation. We next investigated whether the lack of response of FH535 treated cells to LCFA was associated with the downregulation of CPT1α expression levels (Figure 9). Interestingly, we observed an increase in CPT1α expression at both mRNA and protein levels in cells treated with FH535 compared to control or FH535-Me cells. The elevated CPT1α expression suggests that FH535 does not interfere with the mitochondrial LCFA uptake, but it cannot be properly utilized by the ETC to produce ATP.

### Inhibition of glutamine oxidation

Glutamine is the most abundant amino acid in the blood and has a critical role in the synthesis of proteins and nucleic acids and as a precursor of TCA intermediates in proliferating cancer cells. Glutamine is also required in the synthesis of glutathione, a powerful intracellular antioxidant necessary to maintain the cellular redox homeostasis [24–27]. To investigate the role of FH535 in the utilization of glutamine by Huh7 cells, we inhibited the glutamine oxidation pathway using the inhibitor BPTES (Figure 8D). Addition of BPTES reduced the maximal respiration in control (untreated) cells, a finding that indicated the use of glutamine as a substrate for Huh7 cell respiration (Supplementary Figure 1A). However, cells treated with FH535 showed an even lower maximal respiration compared to control (untreated) cells, indicating a higher dependency on glutamine substrate (Figure 8D). The fact that FH535-Me-treated cells showed similar dependency for glutamine compared to FH535-treated cells indicated that this effect was independent of the uncoupling activity of FH535.

**Figure 10:**
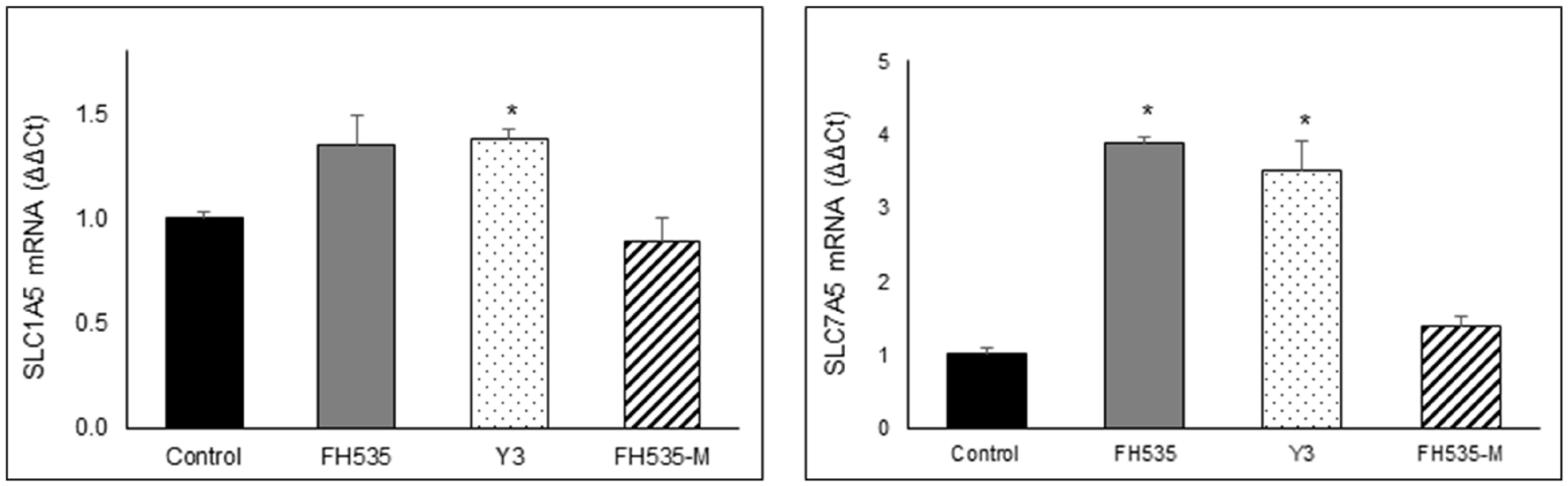
Relative expression of SLC1A5 and SLC7A5 mRNA were determined by real time-PCR after 48 treatment of Huh7 cells with 10 μM of the indicated compounds using B2M gene for normalization. Values are represented as mean ± SD, ^*^ *p* < 0.05 *vs.* control.

The availability of glutamine depends on the interplay of several amino acid transporters that translocate glutamine and glutamate across cell membranes. ASCT2 (*SLC1A5*) and LAT1 (*SLC7A5)* are two transporters commonly over-expressed in cancers [28, 29]. ASCT2 is a Na^+^-dependent amino acid symporter that exchanges glutamine for another amino acid (*i.e*., asparagine, threonine, or serine). In contrast, LAT1 is a Na^+^-independent amino acid antiporter that transports sterically bulky, essential amino acids, such as leucine, phenylalanine or tryptophan, in exchange for intracellular glutamine [26, 28, 29]. We analyzed the expression of these two amino acid transporters, SLC1A5 (ASCT2) and SLC7A5 (LAT1). We observed no consistent change in SLC1A5 (ASCT2) expression after FH535 treatment in Huh7 cells, but an increase in SLC7A5 (LAT1) in FH535- or Y3-treated cells compared to FH-Me or control cells (Figure 10).

## DISCUSSION

HCC tumors display a high level of glucose metabolism that involves increased glucose uptake and lactate production *via* the glycolytic pathway (*i.e*., the Warburg effect) even in the presence of oxygen and functional mitochondrial respiratory machinery. Glutamine is used by cancer cells to increase proliferation as well as survival under metabolic stress conditions. This amino acid plays a critical role as a precursor of glutathione, the most relevant anti-oxidant that counteracts the damaging effects of ROS. Therefore, glutamine pathway represents a potential target in cancer therapeutics. At the same time, lipid catabolism provides NADH and FADH_2_ that in turn drives oxidative phosphorylation and supports the proliferation and growth of cancer cells. Overall, the current proposed model supports the importance of the metabolic reprograming of cancer cells to sustain the unrestrained cell expansion and tumor progression. Our group has defined fundamental mechanisms on the interplay between the Wnt/β-catenin pathway and mitochondrial activity associated with the regulation of the metabolic network in HCC tumor cells. Germane to this interplay, Wnt/β-catenin inhibitors, FH535 and Y3, regulated the fatty acid and glutamine pathways of mitochondrial respiration. These results were consistent with previous studies demonstrating the link between β-catenin signaling and key enzymes of the cellular metabolism in cancer. We previously reported that FH535 and Y3 were inhibitors of the mitochondrial OXPHOS and disrupted the transmembrane potential and the electron transport chain reducing ATP generation [9]. In agreement with other investigators, these results further supported the potential therapeutic benefits of targeting the Wnt pathway and the mitochondria in HCC. We analyzed the mechanism of action of FH535 and Y3 by generating methylated analogs, FH535-Me and Y3-Me, and studied their related effects on metabolic processes. As we described in a recent study [20], FH535 possessed a hydrophobic substructure and an ionizable nitrogen-hydrogen bond that participated in proton translocation in the mitochondrial inner space. After replacing the “active” hydrogen with a methyl group, the methylated analogs, FH535-Me and Y3-Me, lost their activity as proton uncouplers. These methylated derivatives were unable to induce apoptotic cell death or to inhibit cell proliferation in HCC cells.

We demonstrated increased ROS levels after treatment with FH535 and Y3. While ROS is a necessary signaling intermediate for tumor cell growth and activity, an excess of ROS flux induces irreversible changes in multiple macromolecules and contributes to organelle failure, loss of cell integrity, and cell death. Aberrant increase of the endogenous mtROS caused by FH535 triggers stress response mechanisms, including autophagy, to clear damaged macromolecules or organelles and maintain cell homeostasis. However, our previously published results showed that these sulfonamide derivatives—FH535 and Y3—interfered with the autophagic flux and thus prevented the protective mechanisms against oxidative cell damage [17]. In cancer cells, OXPHOS is not completely coupled and a small number of protons flow directly into the mitochondrial matrix across the inner mitochondrial membrane instead of passing through the ATP synthase complex, reducing the efficiency of ATP production. This proton leak phenomenon is enhanced upon FH535 and Y3 treatments and is linked to the functional disruption of the ETC and the uncoupling activity of FH535 in HCC and embryonic kidney cells [18, 20]. Our OCR results in HCC cells showed significant changes in the proton leak upon FH535 or Y3 treatments, but not in the presence of the methylated analogs. These results support the drug-induced mitochondrial dysfunction and, together with the simultaneous increase of the glycolytic rate, suggest a compensatory metabolic shift towards a glycolytic dependency. This rerouting of glucose to glycolysis in FH535-treated cells, in combination with the inability to use LCFA or glutamine as alternative substrates, may further contribute to the progressive functional OXPHOS deterioration induced by sulfonamides in HCC cells.

Depletion of cellular antioxidant defenses, such as glutathione, allows the accumulation of significant quantities of ROS, which is a major trigger of apoptosis. The upregulation of SLC7A5 (LAT1) expression observed after FH535 and Y3 treatments suggests a decrease in the intracellular levels of glutamine affecting glutathione production. Given the critical role of glutamine in the production of glutathione, we can speculate that the increased levels of LAT1 in the presence of FH535 decrease glutathione and allows significant ROS accumulation causing major irreversible HCC cell damage. At the same time, glutamine cannot be properly utilized in the TCA cycle to generate ATP as an alternative substrate. The resulting pro-oxidative state promoted by FH535 and Y3 cause apoptotic cell death and contribute to prevent tumor progression. In a recent study we reported the FH535-induced activation of AMPK and ensuing Acetyl-CoA carboxylase (ACC) inactivation [20]. ACC catalyzes malonyl-CoA synthesis, an inhibitor of CPT1. Therefore, FH535 could promote CPT1 activity. The fact that the expression of CPT1α (but not CD36) is upregulated by FH535 and Y3 indicates that endogenous (but not exogenous) LCFA β-oxidation (but not LCFA transport into mitochondria) is one of the main metabolic pathways altered by these sulfonamides in HCC cancer cells.

In summary, the FH535 and Y3 disruption of mitochondrial redox control in HCC cells resulted from uncoupling mechanisms that increased proton leakage and decreased ATP production leading to apoptotic cell death. The uncoupling effects of the sulfonamides in HCC cells were supported by the loss of activity of the methylated analogs. The accumulation of ROS significantly contributed to cell damage after the impaired autophagic machinery. Lastly, the sulfonamides FH535 and Y3 also targeted glutamine and fatty acid metabolism and caused HCC cells to reprogram their metabolic activity towards the preferential use of glucose and the glycolytic pathway. This was consistent with the previously reported synergistic effect of FH535 in combination with sorafenib, a critical inhibitor of the glycolytic pathway [9]. A detailed understanding of the metabolic alterations induced by small molecules and combination therapies will provide future therapies to treat this difficult neoplasm.

## MATERIALS AND METHODS

### Cell lines

The HCC cell line Huh7 [30] was obtained from Dr. Guangxiang Luo (University of Alabama, Birmingham, AL, USA) and the PLC/PRF/5 cell line was purchased from American Type Culture Collection (ATCC; Manassas, VA, USA). Both cell lines have been recently authenticated by ATCC and were cultured in Dulbecco’s Modified Eagles Medium (DMEM; Gibco, USA) supplemented with 10% fetal bovine serum (FBS; Gibco, USA), non-essential aminoacids (NEAA; Gibco, USA) and penicillin/streptomycin (Gibco, USA).

### Sulfonamides

FH535 was obtained from APExBio; Y3 and other analogs (not used in this study) in order to improve potency/efficacy and to help define their mechanism of action. We also synthetized the N-methylated analogs of FH535 (FH535-Me) and Y3 (Y3-Me) to determine the active component of these molecules and assist in the determination of their mechanism of action, as described by Kril, *et al.* [21] (chemical structures are shown in Figure 1). Stock solutions of these compounds were prepared in dimethyl sulfoxide (DMSO) (vehicle) and sulfonamides were used at the indicated final concentrations in culture medium.

### [^3^H]-Thymidine incorporation assay

Huh7 and PLC/PRF/5 cells were plated in 96-well plates at 3000–4000 cells/well, treated with control vehicle (DMSO) or the concentrations indicated of FH535, FH535-Me, Y3 or Y3-Me and cultured for 72 h. A [^3^H]-thymidine incorporation assay was performed as described [8].

### Apoptosis assay

Apoptosis assay was performed in Huh7 and PLC/PRF/5 cells treated 48 h with DMSO (vehicle control) or with the indicated doses of FH535, Y3, or FH535-M. Cells were harvested and stained with the APC Annexin V apoptosis detection kit with Propidium Iodide (PI) (BioLegend, USA) according to the manufacturing instructions followed by flow cytometry analysis. Flow cytometry data was acquired with an LSRII instrument (BD-Biosciences) and analyzed with FlowJo software (Tree Star).

### Mitochondrial membrane potential

Mitochondrial membrane potential (**Δ**ψ_m_) was determined by tetramethyl-rhodamine ethyl ester (TMRE)-labeling followed by flow cytometry analysis as reported [9].

### Metabolic analysis

Oxygen Consumption Rates (OCR), that serve as an indicator of mitochondrial respiration (OXPHOS), and Extracellular Acidification Rates (ECAR), that serve as an index of glycolysis, were measured on an XFe-96 Extracellular Flux Analyzer (Agilent Technologies), commonly called a Seahorse Bioscience Analyzer, following the protocol and conditions optimized for HCC cells as previously described [9].

The Substrate Oxidation Stress Test Kit (Agilent) was used to determine the reliance or demand of Huh7 cells for three main metabolic substrates: glucose, long-chain fatty acids (LCFA), and glutamine. In this assay, OCR levels are measured before and after adding an inhibitor targeting one of the substrate oxidation pathways following by OCR measurements under conditions of high energy demand induced in a standard mitochondrial stress assay. The inhibitors used for each specific oxidation pathway are as follows: 1, UK5099 that blocks the glucose oxidation pathway by inhibiting mitochondrial pyruvate carrier (MCP) activity and the transport of pyruvate into mitochondria; 2, Etomoxir that blocks the LCFA oxidation pathway by inhibiting carnitine palmitoyltransferase I (CPT1) that is required for LCFA uptake into mitochondria; 3, BPTES that blocks the glutamine oxidation pathway by inhibiting glutaminase 1 (GLS-1) involved in the conversion of the glutamine to glutamate that in turn lowers the levels α-ketoglutarate entering the TCA. These assays were performed by four sequential injections to each well as follows: 1) Port A, Assay media as control or one of the inhibitors mentioned above with following concentrations: 2 μM UK5099, 4 μM Etomoxir, or 3 μM BPTES; 2) Port B, 1 μM Oligomycin; 3) Port C, 0.3 μM FCCP; and 4) Port D, a mixture of 1 μM Rotenone and 1 μM Antimycin A. All reagents used in the Seahorse experiments were purchased from Sigma-Aldrich or Agilent. Analyses of data were performed with Wave 2.6 software (Agilent Technologies, USA), Excel (Microsoft Office 2013) and Prism 7.0 (GraphPad Software).

### Western blot analyses

Cell lysates were prepared in ice-cold, radioimmunoprecipitation assay (RIPA) buffer containing protease inhibitor cocktail (ThermoFisher, Waltham, MA, USA). Protein concentration was determined using the bicinchoninic acid (BCA) Protein Assay (ThermoFisher, Waltham, MA, USA). Cellular proteins (20–40 μg per well) were separated on sodium dodecylsulfate (SDS)-polyacrylamide gel and transferred to polyvinylidene difluoride (PVDF) membrane (ThermoFisher, Waltham, MA, USA). All primary antibodies were purchased from Cell Signaling Technologies (Danvers, MA, USA) and used at 1:1,000 dilution with the exception of the glyceraldehyde 3-phosphate dehydrogenase (GAPDH) antibody, (Thermo Fisher, Waltham, MA, USA) and used at 1:30,000. Proteins were detected by incubating with horseradish peroxidase-conjugated antibodies (Cell Signaling Technologies, Danvers, MA, USA). Specific bands were visualized using enhanced chemiluminescence reagent (BioRad, Hercules, CA, USA) and quantified using the ImageJ software (US NIH, Bethesda, MD, USA).

### Quantitative real-time RT-PCR

Total RNA was extracted with miRNeasy mini kit (Qiagen, Hilden, Germany) and cDNA was produced using iScript cDNA synthesis kit (BioRad, Hercules, CA, USA) from 1 μg of total RNA previously treated with Turbo DNase. Real time quantitative PCR (RT-qPCR) was performed using SsoAdvanced Universal SYBR Green supermix (BioRad, Hercules, CA USA) with specific primers: CPT1α (*CPT1a*: 5′-TCCAGTTGGCTTATCGTGGTG-3′ and 5′- CTAACGAGGGGTCGATCTTGG-3′), ASCT2 (*SLC1A5*: 5′-GTGGCGCTGCGGAAGCT-3′ and 5′- GGCGTACCACATGATCCAG-3′), LAT1 (*SLC7A5*: 5′- CCGTGAACTGCTACAGCGT3′ and 5′- CTTCCCGATCTGGACGAAGC-3′), β2-microglobulin (B2M: 5′-GACTTTGTCACAGCCCAAGATAG-3′ and 5′-TCCAATCCAAATGCGGCATCTTC-3′). Transcript levels were normalized to β-2-macroglobulin level as indicated using the ΔΔCt method.

### Statistical analysis

Data was reported as mean ± SD of triplicate experiments (except where indicated). Statistical analyses were performed using GraphPad PRISM 7.0 (GraphPad Software, San Diego, CA, USA). Statistical significance of differences between two groups was analyzed using Student’s *t*-test or ANOVA with post-hoc Tukey HSD. For the analyses, *p* < 0.05 or *p* < 0.0001 as indicated was considered a statistically significant difference.

## SUPPLEMENTARY MATERIALS


