# Supplementary material for: Mitochondrial uncoupling and the disruption of the metabolic network in hepatocellular carcinoma
Source: Oncotarget. 2020 Aug 4;11(31):3013–24. doi: 10.18632/oncotarget.27680 (PMC7415405; doi:10.18632/oncotarget.27680)
Supplement: Supplementary file 1 [file oncotarget-11-3013-s001.pdf]

Mitochondrial uncoupling and the disruption of the metabolic network in hepatocellular carcinoma

SUPPLEMENTARY MATERIALS

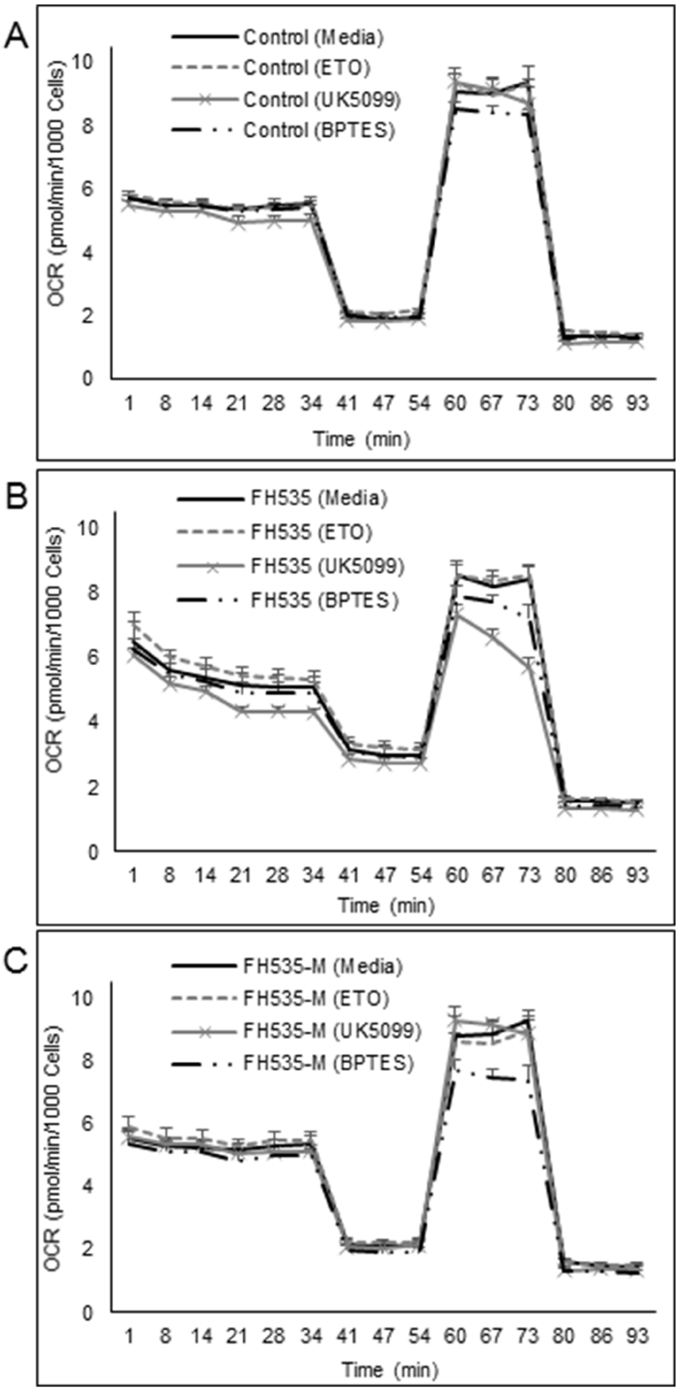

Supplementary Figure 1: Substrate oxidation test after treatment of Huh7 cells for 24 h with DMSO vehicle control or 10 µM of indicated compound as indicated in Figure 8.
